# Non-alcoholic Fatty Liver Disease/Metabolic Dysfunction-Associated Steatotic Liver Disease and the Risk of Incident Atrial Fibrillation: An Updated Systematic Review and Meta-Analysis of Cohort Studies

**DOI:** 10.7759/cureus.117112

**Published:** 2026-09-28

**Authors:** Disha Disha, Allah Rakhio Bhurgri, Deepak Verma, Steve Amirtharaj, Julia Rinaldi Perico, Sandipkumar S Chaudhari, Hijazi Mallick, Neelum Ali

**Affiliations:** 1 Medicine, St. Martinus University, Willemstad, CUW; 2 Medicine, Dow University of Health Sciences, Karachi, PAK; 3 Internal Medicine, Sri Guru Ram Das Institute of Medical Sciences & Research, Amritsar, IND; 4 Internal Medicine, Avalon University School of Medicine, Beckley, USA; 5 Internal Medicine, Nove de Julho University, Sao Paulo, BRA; 6 Cardiothoracic Surgery, University of Alabama at Birmingham, Birmingham, USA; 7 Family Medicine, University of North Dakota School of Medicine and Health Sciences, Fargo, USA; 8 Pediatrics, Dow University of Health Sciences, Karachi, PAK; 9 Internal Medicine, University of Health Sciences, Lahore, PAK

**Keywords:** atrial fibrillation, cohort studies, meta-analysis, metabolic dysfunction-associated steatotic liver disease, nonalcoholic fatty liver disease

## Abstract

Metabolic dysfunction-associated steatotic liver disease (MASLD), formerly non-alcoholic fatty liver disease (NAFLD), has been inconsistently linked to incident atrial fibrillation (AF) across observational cohorts. We performed an updated systematic review and meta-analysis of cohort studies, following the Meta-analysis Of Observational Studies in Epidemiology (MOOSE) guidelines, searching PubMed/MEDLINE, Embase, Scopus, and Web of Science through July 25, 2026, for studies comparing incident AF risk in adults with versus without NAFLD/MASLD. Adjusted effect estimates were pooled using a random-effects model, with heterogeneity assessed via the I² statistic and explored through subgroup and univariable meta-regression analyses. Eighteen cohort studies comprising over 15 million participants from Asia, Europe, and North America met inclusion criteria. Pooling all studies demonstrated a significantly increased risk of incident AF among individuals with NAFLD/MASLD compared with those without (pooled risk ratio (RR) 1.23, 95% confidence interval (CI) 1.12-1.34, p < 0.001), with substantial statistical heterogeneity (I² = 99.4%). In an exploratory analysis of three studies, advanced disease with hepatic fibrosis was also associated with increased AF risk (RR 1.19, 95% CI 1.11-1.28). In the present meta-analysis, NAFLD/MASLD was associated with a modestly higher risk of incident AF after adjustment for conventional cardiometabolic risk factors, although heterogeneity was very high. These findings support attention to AF risk in this population and the use of standardized diagnostic approaches in future research.

## Introduction and background

Fatty liver disease unrelated to excessive alcohol intake has been described under three successive nomenclatures. Non-alcoholic fatty liver disease (NAFLD) was defined by hepatic steatosis in the absence of significant alcohol consumption and other secondary causes of liver fat [1]. In 2020, metabolic dysfunction-associated fatty liver disease (MAFLD) was proposed as a positive diagnosis. It requires hepatic steatosis plus overweight/obesity, type 2 diabetes, or at least two metabolic risk abnormalities, and does not exclude concomitant alcohol use or other liver diseases [2]. In 2023, a multisociety Delphi consensus introduced metabolic dysfunction-associated steatotic liver disease (MASLD). MASLD is defined as hepatic steatosis with at least one of five cardiometabolic risk factors and limited alcohol intake, and a separate category (MetALD) covers individuals with greater alcohol consumption [3]. These definitions identify overlapping but not identical populations. However, approximately 99% of individuals meeting NAFLD criteria also fulfil MASLD criteria [4], which supports combining NAFLD- and MASLD-based cohorts in a single synthesis. MAFLD diverges more substantially because it can include individuals with significant alcohol intake. Once regarded primarily as a hepatic condition with a trajectory toward steatohepatitis, fibrosis, cirrhosis, and hepatocellular carcinoma, MASLD is now recognized as a systemic metabolic disorder with substantial extrahepatic consequences [5]. Cardiovascular disease, rather than liver-related complications, is the leading cause of death among individuals with MASLD, and mounting evidence implicates the condition in a broad spectrum of cardiac structural and electrical abnormalities, including left ventricular hypertrophy, diastolic dysfunction, and cardiac arrhythmias [4,5].

Atrial fibrillation (AF), the most common sustained cardiac arrhythmia encountered in clinical practice, carries a well-established burden of stroke, heart failure, and mortality, with healthcare costs that continue to escalate as global prevalence rises [6,7]. Because MASLD and AF share overlapping risk factors including obesity, insulin resistance, type 2 diabetes, and hypertension, a growing number of observational cohort studies have investigated whether MASLD independently predicts incident AF, beyond these shared metabolic drivers [8-10]. Proposed mechanistic links include chronic low-grade systemic inflammation, ectopic epicardial and pericardial fat deposition, autonomic dysfunction, and insulin resistance-driven atrial structural remodeling [5,9,11].

Despite this biological plausibility, epidemiological findings have been inconsistent. Early studies, largely restricted to patients with type 2 diabetes or small single-center cohorts, reported markedly elevated risk estimates [8,12], while larger population-based cohorts from Europe and North America have shown more modest or even null associations [13,14]. Several meta-analyses have attempted to reconcile these discrepancies. Earlier pooled analyses combined cross-sectional, case-control, and cohort designs, limiting causal inference [15,16]. More recent updates restricted to cohort studies have consistently found a modest but statistically significant increase in AF risk associated with NAFLD/MASLD [17,18]. However, both analyses were limited by substantial unexplained heterogeneity, incomplete geographic representation, and restricted assessment of dose-response relationships according to disease severity or fibrosis stage [17,18].

Since these prior syntheses, several large nationwide cohort studies have been published, including analyses stratifying risk by hepatic fibrosis severity, age group, and diabetes status, as well as studies applying the revised MASLD nomenclature and steatotic liver disease subclassification system [19,20]. These additions substantially expand the available evidence base and allow for more granular exploration of whether disease severity, diagnostic modality, or population characteristics modify the NAFLD/MASLD-AF relationship.

Given the persistent clinical uncertainty, the evolving nomenclature from NAFLD to MASLD, and the accumulation of new cohort data since the most recent systematic reviews, an updated and comprehensive meta-analysis is warranted. The present study aimed to quantify the pooled risk of incident AF among adults with MASLD across all available cohort studies. Compared with the most recent cohort-only meta-analyses [17,18], the present review included additional recently conducted studies. Additionally, it examines whether the association differs by diagnostic modality (imaging, biomarker indices, diagnostic codes, or biopsy) and by geographic region. Finally, it uses meta-regression to test whether patient-level characteristics (age, sex, diabetes, and hypertension prevalence) and study-level characteristics (follow-up duration, publication year) explain the substantial between-study heterogeneity reported previously.

## Review

Methodology

We performed this systematic review and meta-analysis of observational studies following the Meta-analysis Of Observational Studies in Epidemiology (MOOSE) guidelines [21].

Literature Search and Search Strategy

A comprehensive literature search was performed across the following electronic databases: PubMed/MEDLINE, Embase, Scopus, and Web of Science from database inception to July 25, 2026. No language or publication-date restrictions were applied at the search stage. The search strategy combined Medical Subject Headings (MeSH) terms and free-text keywords connected using the Boolean operators "AND" and "OR." Search terms for the exposure included "non-alcoholic fatty liver disease," "NAFLD," "metabolic dysfunction-associated steatotic liver disease," "MASLD," "metabolic dysfunction-associated fatty liver disease," "MAFLD," "hepatic steatosis," "fatty liver," "atrial fibrillation," "AF," and "arrhythmia." The reference lists of all included studies and relevant prior systematic reviews and meta-analyses on this topic were manually screened (backward citation searching) to identify additional eligible studies not captured by the electronic search. The search was performed by two authors independently. Any disagreement between the two authors was resolved through discussion. The detailed search strategy is provided in the appendix.

Study Selection

Titles and abstracts identified through the search strategy were screened independently against the pre-specified eligibility criteria, followed by full-text review of potentially eligible articles. Studies were included if they (1) enrolled adult human participants; (2) compared individuals with NAFLD or MASLD (diagnosed by any of imaging, blood-biomarker indices such as the fatty liver index, diagnostic codes, or liver biopsy) against a non-NAFLD/MASLD comparator group; (3) reported incident (new-onset) AF as an outcome, ascertained during longitudinal follow-up; and (4) reported an adjusted or unadjusted hazard ratio (HR), risk ratio (RR), or odds ratio with a corresponding 95% confidence interval (CI), or provided sufficient raw data to calculate one.

Studies were excluded if they reported only prevalent (cross-sectional) AF without a longitudinal incident AF analysis, used a continuous liver-biomarker measure without a defined NAFLD/MASLD-versus-comparator contrast, enrolled a population selected on the basis of pre-existing AF or another cardiac condition rather than at risk of incident AF, or did not report an extractable effect estimate for the NAFLD/MASLD-AF association specifically.

Two reviewers independently screened the titles and abstracts of all identified records and retrieved the full texts of studies considered potentially eligible. Each full-text article was then assessed independently by both reviewers to determine its compliance with the predefined inclusion and exclusion criteria. Any disagreements regarding study eligibility were resolved through discussion, and when necessary, a third reviewer was consulted to reach a final consensus.

Data Extraction

For each eligible study, the following data were extracted independently using a standardized, piloted extraction form: first author and publication year, country/region of study, study design, number of subjects with NAFLD/MASLD and comparators, follow-up duration, mean age, number of males, method used to diagnose NAFLD/MASLD, number of subjects with diabetes and hypertension, and outcome data. Where a study reported multiple models with progressively greater covariate adjustment, the most fully adjusted model was extracted as the primary estimate. Data were extracted by two authors. Discrepancies in data extraction were resolved by re-review of the original source text.

Quality Assessment

The methodological quality and risk of bias of the included cohort studies were evaluated using the Newcastle-Ottawa Scale (NOS) [22]. The NOS assesses the quality of observational cohort studies across three main domains: selection, comparability, and outcome. The selection domain evaluates the representativeness of the exposed cohort, appropriateness of the non-exposed cohort, ascertainment of exposure, and confirmation that the outcome of interest was absent at baseline. The comparability domain assesses the extent to which potential confounding factors were appropriately controlled through study design or statistical adjustment. The outcome domain evaluates the methods used for outcome ascertainment, adequacy of the follow-up period, and completeness of cohort follow-up.

Data Analysis

The primary outcome of interest was the risk of incident AF among adults with and without MASLD during the follow-up period. RRs with corresponding 95% CIs were used as the measures of effect for individual studies.

Statistical heterogeneity across the included studies was assessed using Cochran’s chi-squared (Q) test and the I² statistic. The I² statistic quantifies the proportion of total variability in the observed effect estimates that is attributable to between-study heterogeneity rather than sampling error. An I² value greater than 50% was considered indicative of substantial heterogeneity.

Subgroup analyses and meta-regression were conducted to investigate potential sources of heterogeneity and assess the robustness of the observed association. Subgroup analyses were performed according to study country, the methodology used to diagnose MASLD, and the severity of MASLD. In addition, univariable meta-regression analyses were performed to examine whether specific study-level moderator variables contributed to the observed between-study heterogeneity.

All statistical tests were two-sided, with a p-value < 0.05 considered statistically significant. All analyses were performed using RevMan (The Cochrane Collaboration, London, UK) and R statistical software (R Foundation for Statistical Computing, Vienna, Austria) with the meta and metafor packages.

Results

Figure 1 illustrates the PRISMA flow diagram outlining the study selection process for the meta-analysis. Following the screening of titles and abstracts and the removal of duplicate records, 18 studies were deemed eligible and included in the meta-analysis. The characteristics of the included studies are presented in Table 1.

**Figure 1:**
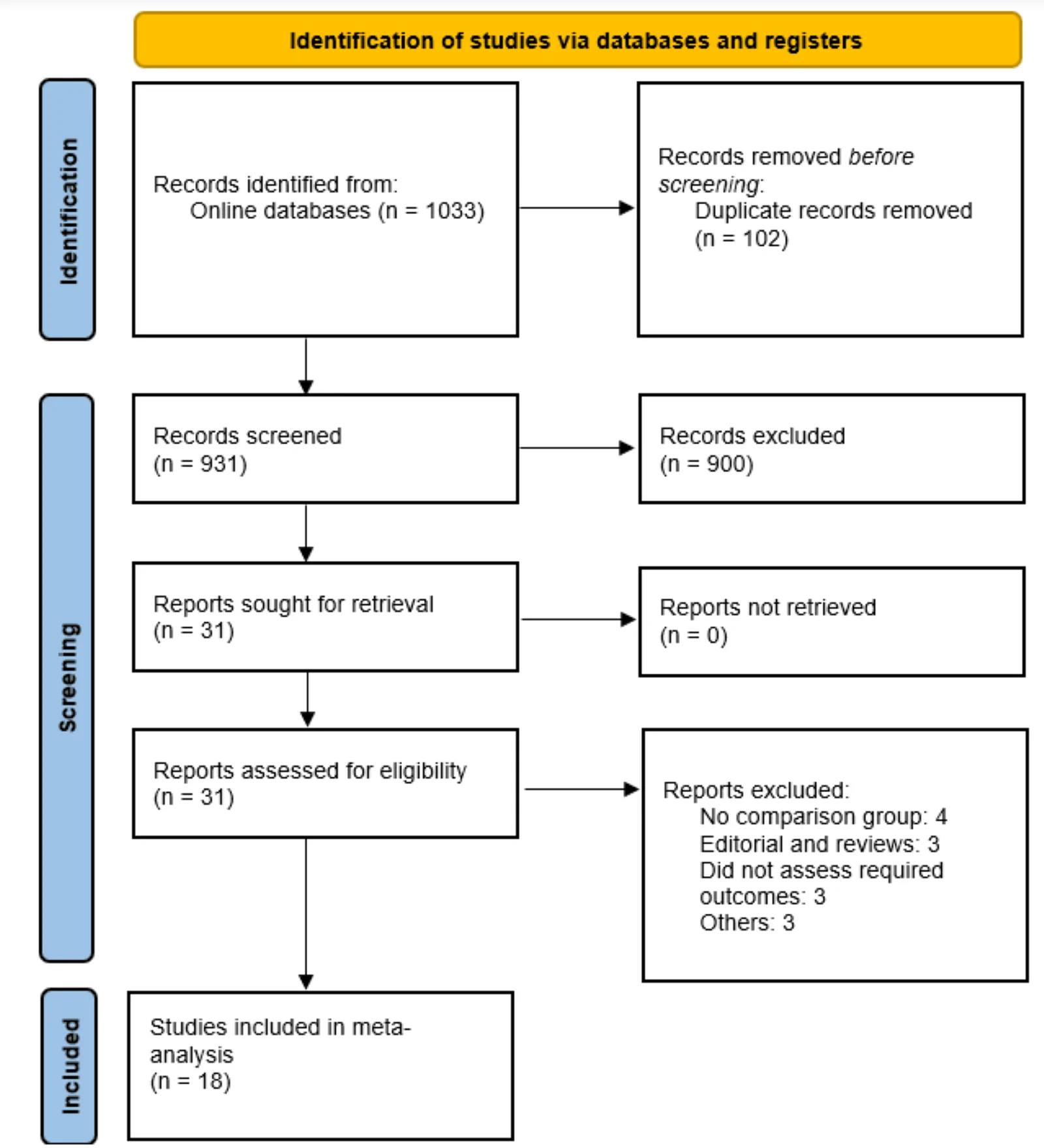
Study selection process

**Table 1:** Characteristics of included studies FLI: fatty liver index; ICD: International Classification of Diseases; MDCT: multidetector computed tomography; NAFLD: non-alcoholic fatty liver disease; MASLD: metabolic dysfunction-associated steatotic liver disease

Study	Region	Design	Diagnosis of NAFLD/MASLD	Follow-up	Mean age (years)	Number of males (n)	NAFLD (n)	Control (n)
Allen et al., 2019 [12]	United States	Retrospective cohort	ICD-9	7 years	53.0	9,073	3,869	15,209
Baratta et al., 2020 [23]	Italy	Prospective cohort	Ultrasound	3.45 years	56.4	561	643	255
Cho et al., 2024 [19]	South Korea	Retrospective population-based cohort	FLI	9.3 years	46.7	1,639,453	1,075,360	2,104,222
Cho et al., 2025 [20]	South Korea	Retrospective population-based cohort			56.5	1,317,392	510,905	1,778,355
van Kleef et al., 2022 [13]	Netherlands	Prospective population-based cohort	Ultrasound		69.5	2,499	2,079	3,746
Käräjämäki et al., 2015 [10]	Finland	Prospective cohort	Ultrasound	16.3 years	51.3	450	249	709
Labenz et al., 2020 [24]	Germany	Retrospective cohort		10 years	55.6	22,136	22,048	22,048
Lee et al., 2021 [25]	South Korea	Retrospective population-based cohort	FLI	8.3 years	46.7	4,184,989	2,738,621	5,309,434
Lei et al., 2022 [26]	China	Retrospective longitudinal cohort	Ultrasound-diagnosed steatosis + metabolic dysfunction criteria	2.22 years	NR	NR	18,286	36,667
Long et al., 2017 [14]	United States	Prospective community-based cohort	Hepatic steatosis by MDCT	9.3 years	59.0	1,002	423	1,699
Park et al., 2025 [27]	South Korea	Retrospective population-based cohort	FLI	10.6 years	30.9	3,477,036	1,448,817	4,603,095
Roh et al., 2020 [28]	South Korea	Retrospective population-based cohort	FLI	5.3 years	40.2	79,477	83,575	83,558
Simon et al., 2023 [29]	Sweden	Population-based matched cohort	Liver biopsy-confirmed	10.8 years	53.0	34,195	11,206	51,856
Tang et al., 2025 [30]	China	Retrospective cohort	Hepatic ultrasound	3.65 years	73.0	5,153	3,926	4,585
Targher et al., 2013 [8]	Italy	Prospective cohort	Hepatic ultrasound	10 years	63.5	235	281	119
You et al., 2016 [31]	South Korea	Retrospective population-based cohort	FLI	3.7 years	49.0	84,571	107,619	125,360
Zhai et al., 2026 [32]	United Kingdom	Retrospective population-based cohort	FLI	12.88 years	53.0	44,071	30,972	61,609
Zou et al., 2021 [33]	United Kingdom	Prospective cohort	FLI	7.86 years	56.6	92,901	66,164	129,964

A total of 18 cohort studies, published between 2013 and 2026 and comprising over 15 million participants, met the inclusion criteria and were included in this meta-analysis (Table 1). Studies originated from Asia (n = 8), Europe (n = 8), and North America (n = 2). MASLD was diagnosed using the fatty liver index or other non-invasive biomarker scores in eight studies, imaging (ultrasound or computed tomography) in seven studies, diagnostic/administrative codes in two studies, and liver biopsy in one study. Study designs included retrospective and prospective population-based cohorts, with follow-up durations ranging from 2.2 to 16.3 years. Mean participant age across studies ranged from 30.9 to 73.0 years, and the proportion of male participants ranged from approximately 36% to 61%. Study populations included general adult cohorts, individuals with type 2 diabetes, and age-restricted (young adult and elderly) populations. Full details of study-level characteristics, including region, design, diagnostic method, follow-up duration, mean age, number of male participants, sample size, and adjusted effect estimates for each study, are presented in Table 1. Table 2 presents the quality assessment of included studies.

**Table 2:** Quality assessment of included studies

Study	Selection	Comparability	Outcome	Total	Quality
Allen et al., 2019 [12]	4	1	4	9	High
Baratta et al., 2020 [23]	2	1	2	5	Moderate
Cho et al., 2024 [19]	4	2	3	9	High
Cho et al., 2025 [20]	4	2	3	9	High
Käräjämäki et al., 2015 [10]	3	2	3	8	High
van Kleef et al., 2022 [13]	3	2	2	7	High
Labenz et al., 2020 [24]	4	2	3	9	High
Lee et al., 2021 [25]	4	1	4	9	High
Lei et al., 2022 [26]	3	2	2	7	High
Long et al., 2017 [14]	3	2	3	8	High
Park et al., 2025 [27]	4	2	3	9	High
Roh et al., 2020 [28]	3	2	3	8	High
Simon et al., 2023 [29]	4	2	3	9	High
Tang et al., 2025 [30]	3	2	2	7	High
Targher et al., 2013 [8]	3	2	3	8	High
You et al., 2016 [31]	3	1	2	6	Moderate
Zhai et al., 2026 [32]	4	2	3	9	High
Zou et al., 2021 [33]	4	2	3	9	High

Risk of Incident AF in Patients With NAFLD/MASLD

A total of 18 studies, comprising over 15 million participants, reported data on the association between NAFLD/MASLD and incident AF and were included in the pooled analysis. Pooling all 18 studies using a random-effects model demonstrated that patients with NAFLD/MASLD had a significantly increased risk of incident AF compared with individuals without NAFLD/MASLD (pooled RR 1.23; 95% CI 1.12-1.34; p < 0.001). Substantial statistical heterogeneity was observed across the included studies (I² = 99%), as shown in Figure 2. Despite this heterogeneity, the overall pooled estimate remained statistically significant and directionally consistent with the hypothesis that NAFLD/MASLD is associated with an increased risk of incident AF. Figure 3 presents the funnel plot. Funnel plot inspection suggested mild visual asymmetry, but neither Egger's regression test (intercept −0.79, 95% CI −8.74 to 7.16, p = 0.836) nor Begg's rank correlation test (Kendall's tau = 0.079, p = 0.649) found statistically significant evidence of small-study effects. Given the substantial between-study heterogeneity (I² = 99%), these results should be interpreted cautiously, as both asymmetry and heterogeneity can independently distort funnel plot appearance.

**Figure 2:**
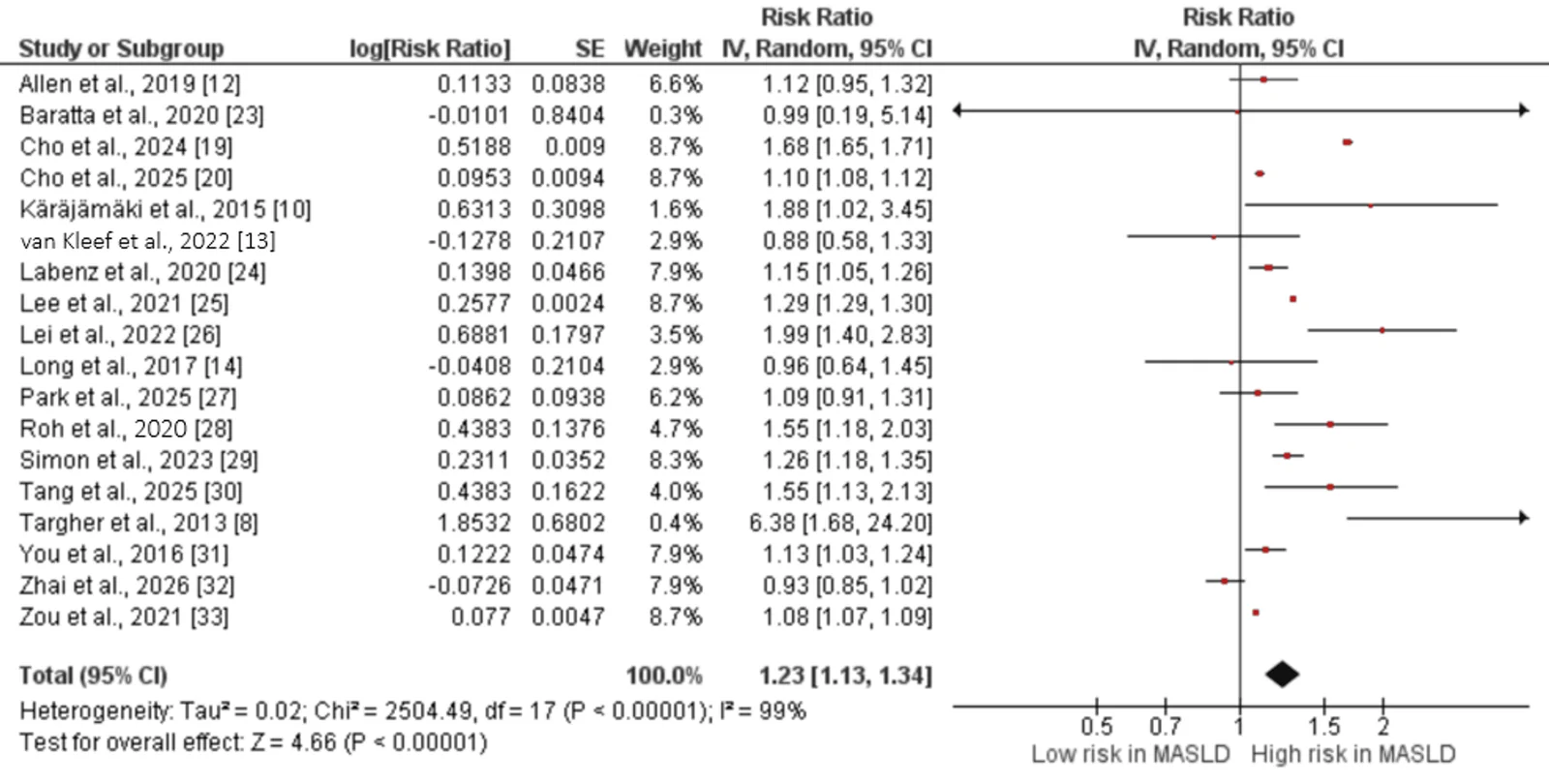
Risk of AF in MASLD subjects AF: atrial fibrillation; MASLD: metabolic dysfunction-associated steatotic liver disease; CI: confidence interval References: [8,10,12-14,19,20,23-33]

**Figure 3:**
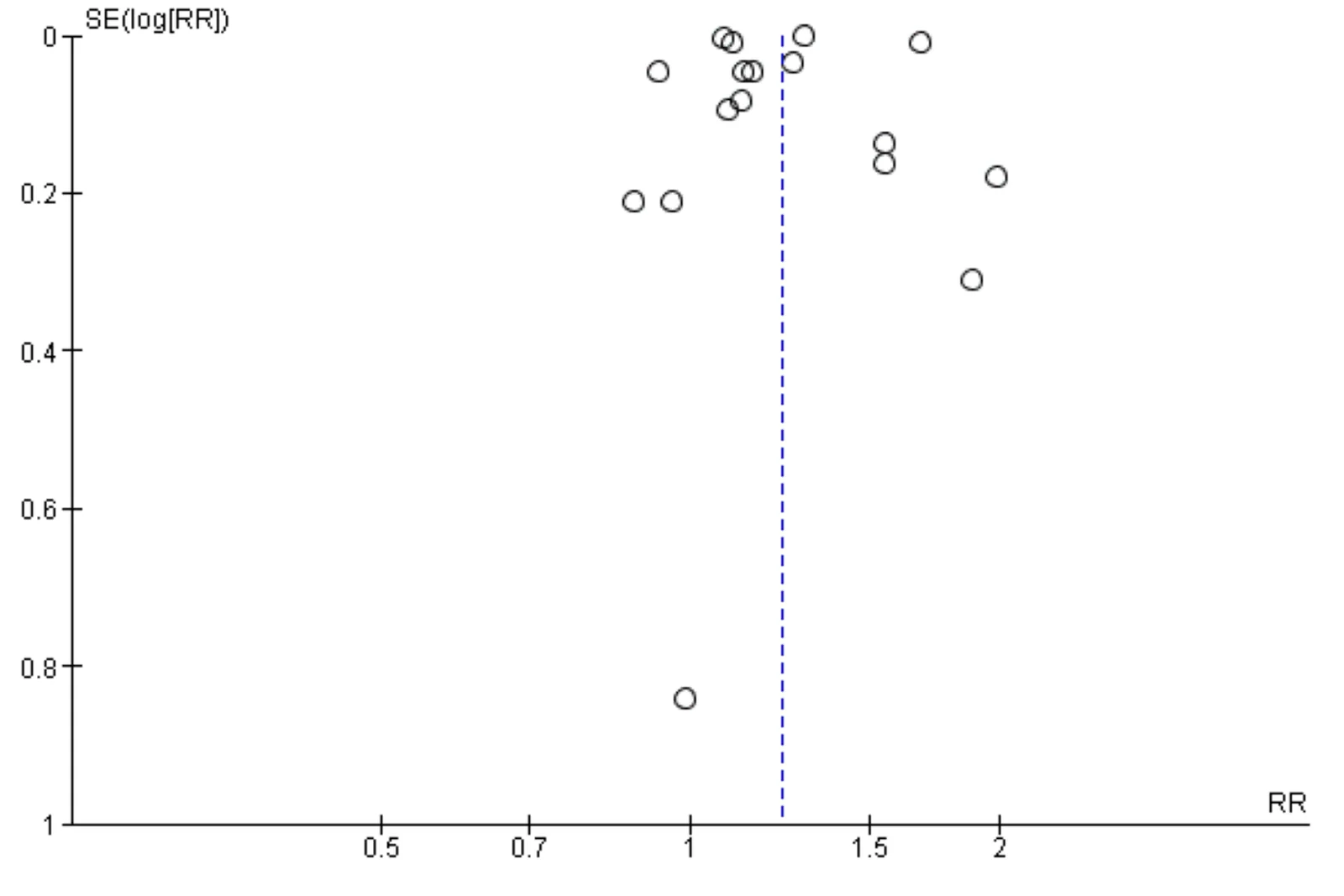
Funnel plot (publication bias) RR: risk ratio

Subgroup Analysis

Table 3 presents subgroup analyses of the association between NAFLD/MASLD and incident AF, stratified by continent and by diagnostic method. Pooled random-effects RRs were higher in Asian cohorts (RR 1.34) than in European (RR 1.11) or North American (RR 1.10) cohorts. Across diagnostic methods, imaging-based studies showed the strongest association (RR 1.46), followed by biopsy, FLI, and ICD-10-based diagnoses. Substantial heterogeneity persisted within most subgroups, particularly those defined by Asian region and FLI-based diagnosis.

**Table 3:** Subgroup analysis of the association between NAFLD/MASLD and incident atrial fibrillation, by continent and diagnostic method CI: confidence interval; FLI: fatty liver index; ICD-10: International Classification of Diseases, 10th revision; I²: heterogeneity statistic; k: number of studies; MASLD: metabolic dysfunction-associated steatotic liver disease; RR: risk ratio; NAFLD: non-alcoholic fatty liver disease

Subgroup	Categories	k	RR (95% CI)	I²	p-value
Continent	USA [12,14]	2	1.10 (0.94-1.28)	0.00%	0.04
	Europe [8,10,13,23,24,29,32,33]	8	1.11 (1.01-1.23)	83.20%	
	Asia [19,20,25-28,30,31]	8	1.34 (1.18-1.51)	99.60%	
Diagnosis of MASLD	Imaging [8,10,13,14,23,26,30]	7	1.46 (1.04-2.06)	67.60%	
	FLI [19,20,25,27,28,31-33]	8	1.20 (1.07-1.34)	99.80%	0.21
	ICD-10 [12,24]	2	1.14 (1.05-1.24)	0.00%	
	Biopsy [29]	1	1.26 (1.18-1.35)	NA (k = 1)	

Advanced MASLD and Risk of Incident AF

Advanced MASLD/NAFLD, defined by severe hepatic fibrosis on either non-invasive biomarker scores (BARD score) or liver biopsy, was distributed across three cohort studies examining its association with incident AF. Compared with individuals without MASLD/NAFLD, those with advanced disease demonstrated a significantly elevated risk of developing AF (n = 3 studies; random-effects HR 1.19, 95% CI 1.11-1.28; I² = 87.4%). This risk appeared somewhat more pronounced in the cohort relying on liver biopsy for fibrosis staging (HR 1.59, 95% CI 1.15-2.19) relative to the two cohorts using non-invasive fibrosis scoring (HR 1.14-1.22). Nonetheless, given the marked heterogeneity across studies (I² = 87.4%) and the differing severity thresholds used to define "advanced" disease, these findings should be interpreted as suggestive rather than confirmatory of a clear dose-dependent gradient with fibrosis severity.

Meta-Regression

The results of univariable meta-regression analyses examining the impact of potential moderator variables did not show any significant effect of mean age (p = 0.570), male sex proportion (p = 0.258), follow-up duration (p = 0.156), publication year (p = 0.494), diabetes prevalence (p = 0.978), or hypertension prevalence (p = 0.953) on the association between NAFLD/MASLD and the risk of incident AF (Table 4). None of these six moderators reached statistical significance, so these R² values should not be interpreted as evidence of a true explanatory effect (Figure 4).

**Table 4:** Meta-regression k: number of studies; CI: confidence interval

Moderator	k	Coefficient	95% CI	p-value	R² (%)
Mean age (years) [8,10,12-14,19,20,23-25,27-33]	17	−0.0026	−0.0117 to 0.0064	0.57	20.5
Male sex (%) [8,10,12-14,19,20,23-25,27-33]	17	0.0094	−0.0069 to 0.0258	0.258	0
Follow-up duration (years) [8,10,12,14,19,23-33]	16	−0.0202	−0.0482 to 0.0077	0.156	0
Publication year [8,10,12-14,19,20,23-33]	18	−0.0110	−0.0426 to 0.0206	0.494	0
Diabetes prevalence (%) [8,12,13,19,20,23-30,32,33]	15	−0.0000	−0.0035 to 0.0034	0.978	0
Hypertension prevalence (%) [8,12,13,19,20,23-30,32,33]	15	−0.0001	−0.0047 to 0.0045	0.953	0

**Figure 4:**
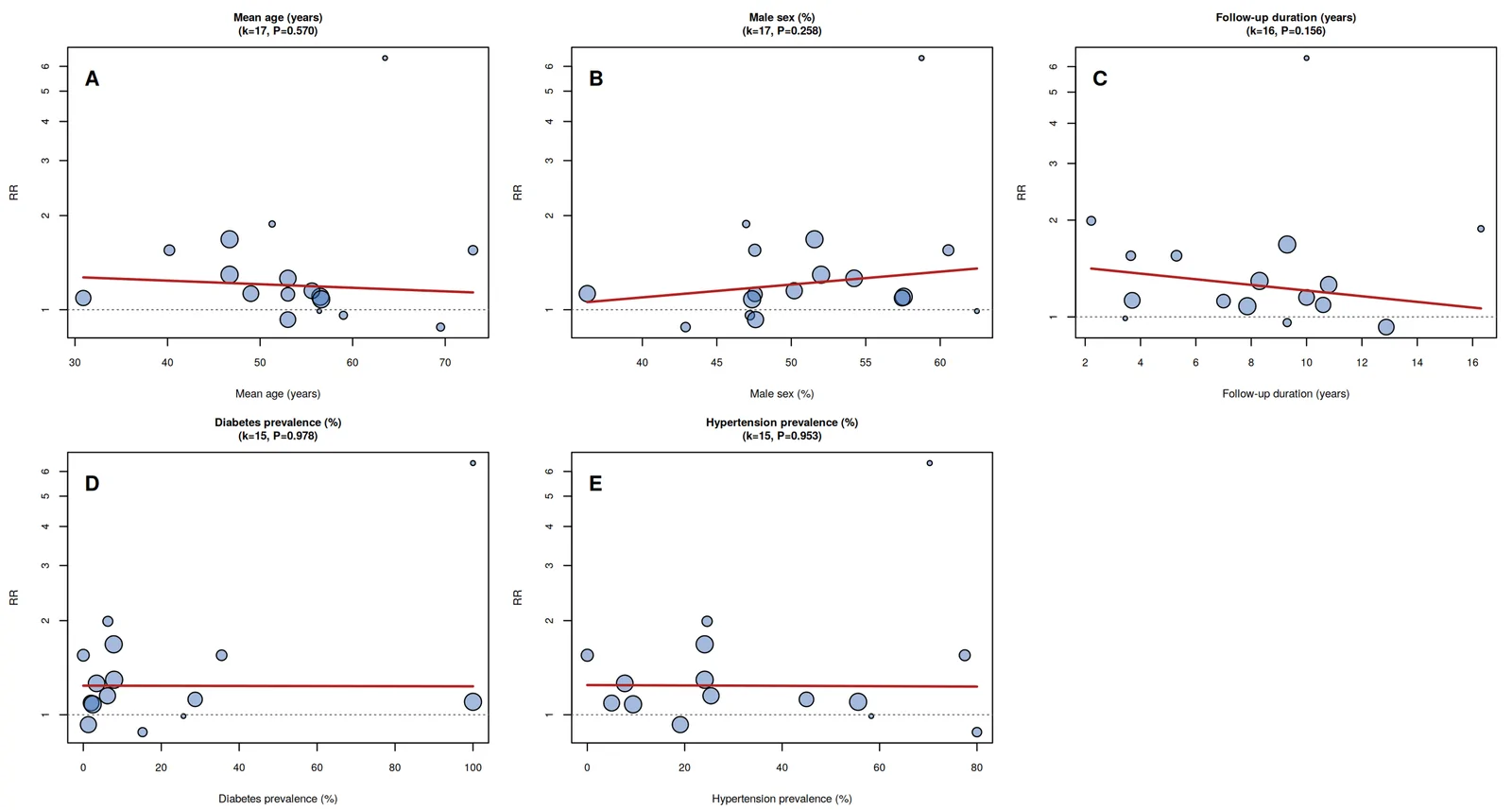
Bubble plots of univariable meta-regression analyses examining the association between study-level moderators and the pooled effect estimate (RR) for incident atrial fibrillation in NAFLD/MASLD Each panel plots individual studies against one moderator: (A) mean age, (B) male sex proportion, (C) follow-up duration, (D) diabetes prevalence, and (E) hypertension prevalence. The y-axis (RR) represents the RR reported by each study, combined on the log scale. The red line represents the fitted meta-regression slope; the dotted gray horizontal line marks the null effect (RR = 1.0). None of the moderators tested reached statistical significance (all p > 0.05). RR: risk ratio; NAFLD: non-alcoholic fatty liver disease; MASLD: metabolic dysfunction-associated steatotic liver disease

Discussion

In this meta-analysis of 18 cohort studies, NAFLD/MASLD was associated with a 23% higher risk of incident AF compared with the absence of NAFLD/MASLD (pooled RR 1.23, 95% CI 1.12-1.34). Because all included studies were observational and heterogeneity was very high, this estimate should be interpreted as an average association across diverse populations and diagnostic approaches, not as evidence of a causal effect. The finding is nevertheless consistent with the recognition of NAFLD/MASLD as a systemic metabolic disorder with potential cardiac consequences [3-5].

Our pooled estimate is broadly consistent with, though modestly higher than, those reported in earlier cohort-restricted meta-analyses. Zhou et al. [17], synthesizing 13 cohort studies with 14.3 million participants, reported a pooled HR of 1.18 (95% CI 1.12-1.23), while the most recent update by Mantovani et al. [18], incorporating 16 cohorts and approximately 19.5 million individuals, reported a similar estimate of 1.20 (95% CI 1.10-1.32). Earlier meta-analyses that pooled cross-sectional, case-control, and cohort designs together reported considerably larger effect sizes, with odds ratios ranging from approximately 1.7 to 2.5 [15,16]; however, these estimates are difficult to interpret causally given the inclusion of prevalent-case designs susceptible to reverse causation and detection bias. By restricting inclusion to cohort studies with incident AF as the outcome, our analysis - consistent with Zhou et al. and Mantovani et al. - provides a more temporally coherent estimate of risk, and the slightly higher pooled effect observed here may reflect the incorporation of newer, larger-scale nationwide cohorts with more granular case ascertainment and longer follow-up [16,17].

Several biologically plausible mechanisms have been proposed to explain the association between NAFLD/MASLD and incident AF, and most converge on the concept of NAFLD/MASLD as both a marker and a mediator of systemic cardiometabolic dysfunction. First, NAFLD/MASLD is characterized by chronic low-grade systemic inflammation, with hepatic steatosis promoting oxidative stress and the release of pro-inflammatory adipokines such as tumor necrosis factor-α, interleukin-6, and C-reactive protein; elevated circulating levels of these inflammatory markers have been independently linked to the initiation, perpetuation, and recurrence of AF [9,11,34]. Second, NAFLD/MASLD is associated with increased ectopic fat deposition in myocardial, epicardial, and pericardial depots; because epicardial adipose tissue lies in direct anatomical proximity to the atrial myocardium without an intervening fascial barrier, it can secrete adipo-fibrokines that directly promote atrial fibrosis and electrical instability [35]. Third, NAFLD/MASLD has been associated with cardiac autonomic dysfunction in population-based studies, altering the sympathovagal balance implicated in AF initiation and maintenance [36]. Fourth, shared insulin resistance and metabolic dysregulation likely contribute to atrial structural remodeling independent of overt diabetes, offering a plausible explanation for why the association persists even in cohorts not restricted to diabetic populations [5,20]. These mechanisms provide biological plausibility for the observed association. However, the present data cannot determine whether NAFLD/MASLD contributes directly to AF or is primarily a marker of shared upstream metabolic dysfunction.

A notable feature of the present analysis was the very high statistical heterogeneity observed across studies (I² = 99.4%) [37], a finding also reported in both prior cohort-restricted meta-analyses [17,18]. Meta-regression analysis in the present study did not identify mean age, sex distribution, follow-up duration, publication year, or the prevalence of diabetes or hypertension as significant moderators of the pooled effect. However, meta-regression with 15 to 18 studies has limited power, and study-level averages may not capture patient-level effect modification. The absence of significant moderators therefore does not exclude a contribution of population characteristics to the observed heterogeneity. This pattern mirrors the subgroup findings of Zhou et al. [17], who similarly found that clinical and demographic subgroups did not fully account for the observed heterogeneity, while methodological factors - particularly the diagnostic modality used to define NAFLD (imaging versus fatty liver index versus diagnostic codes) and the method of AF ascertainment (electrocardiography versus administrative coding versus medical record review) - appeared more influential. Taken together with our findings, this suggests that the heterogeneity in this literature is more likely attributable to differences in study design and outcome/exposure measurement than to differences in the underlying patient populations, an important distinction for interpreting the clinical applicability of the pooled estimate.

The analysis of advanced disease should be regarded as exploratory. Only three studies contributed, and they defined advanced fibrosis differently (BARD score or histology). Each compared advanced disease with the absence of NAFLD/MASLD rather than with milder NAFLD/MASLD, and the pooled estimate (RR 1.19, 95% CI 1.11-1.28) did not exceed that of the overall analysis. The higher estimate in the biopsy-based cohort [29] is compatible with a severity gradient but does not demonstrate one. Whether AF risk increases with fibrosis stage requires studies that directly compare fibrosis stages within the same population.

This meta-analysis has several limitations. First, as with all meta-analyses of observational cohort studies, causality cannot be established, and residual or unmeasured confounding - including variation in alcohol consumption, physical activity, and genetic predisposition - cannot be excluded despite adjustment for major cardiometabolic risk factors in most included studies. Second, the substantial heterogeneity (I² = 99.4%) persisted despite extensive subgroup and meta-regression analysis, and although this argues against clinical population differences as the primary driver, it nonetheless limits the precision and generalizability of the single pooled estimate and suggests that methodological heterogeneity across studies remains incompletely characterized. Third, diagnostic ascertainment of NAFLD/MASLD varied considerably across studies, ranging from ultrasonography and computed tomography to fatty liver index and administrative diagnostic codes, each with differing sensitivity and specificity for detecting hepatic steatosis, particularly in mild disease [2]; this heterogeneity in exposure classification may have introduced misclassification bias in either direction. Fourth, few included studies reported outcomes stratified by fibrosis severity, limiting our ability to fully characterize a dose-response relationship between disease severity and AF risk, despite biological plausibility for such a gradient [19]. Fifth, most large-scale cohorts were derived from Asian, and to a lesser extent European and North American, administrative databases, which may limit generalizability to other populations and healthcare settings.

From a clinical practice perspective, these findings support increased vigilance for incident AF among individuals with NAFLD/MASLD, particularly as the diagnosis becomes more frequently and incidentally identified through routine imaging and non-invasive fibrosis scoring in primary care and hepatology settings. Given that NAFLD/MASLD and AF share overlapping cardiometabolic risk factors, clinicians managing patients with NAFLD/MASLD may consider this association when assessing overall cardiovascular risk, particularly in the context of guideline-directed management of coexisting obesity, diabetes, and hypertension. For research, the persistent unexplained heterogeneity underscores the need for future primary studies to adopt standardized, ideally imaging- or biopsy-confirmed, diagnostic criteria for NAFLD/MASLD alongside validated, prospectively ascertained AF outcomes, rather than relying solely on administrative coding. Further prospective cohorts with longer follow-up and dedicated fibrosis-stratified analyses are needed to clarify whether AF risk escalates with advancing liver disease severity, as suggested by preliminary evidence [19], and mechanistic studies are warranted to establish whether the observed association reflects a direct causal pathway or a shared upstream metabolic driver amenable to common therapeutic intervention.

## Conclusions

In this updated meta-analysis of 18 observational cohort studies, NAFLD/MASLD was associated with a modestly higher incidence of AF, with a pooled relative risk of 1.23. This association persisted after adjustment for conventional cardiometabolic risk factors in the included studies. However, residual confounding cannot be excluded, and causality cannot be inferred. Heterogeneity between studies was very high. It was not explained by study-level differences in age, sex, follow-up duration, or comorbidity burden, and may partly reflect variation in how NAFLD/MASLD and AF were defined and ascertained. An exploratory analysis of advanced fibrosis, based on three studies, did not establish whether AF risk increases with liver disease severity. Clinicians managing patients with NAFLD/MASLD may consider AF within the broader assessment of cardiovascular risk. Future cohort studies should use standardized diagnostic criteria, validated AF ascertainment, and direct comparisons across fibrosis stages.

## Appendices

**Table 5:** Search strategies

Database	Search strategy
PubMed/MEDLINE	("Non-alcoholic Fatty Liver Disease"[Mesh] OR "Fatty Liver"[Mesh] OR "non-alcoholic fatty liver disease"[tiab] OR "nonalcoholic fatty liver disease"[tiab] OR NAFLD[tiab] OR "metabolic dysfunction-associated steatotic liver disease"[tiab] OR MASLD[tiab] OR "metabolic dysfunction-associated fatty liver disease"[tiab] OR "metabolic associated fatty liver disease"[tiab] OR MAFLD[tiab] OR "hepatic steatosis"[tiab] OR "fatty liver"[tiab] OR "steatotic liver disease"[tiab]) AND ("Atrial Fibrillation"[Mesh] OR "Arrhythmias, Cardiac"[Mesh] OR "atrial fibrillation"[tiab] OR AF[tiab] OR arrhythmia*[tiab])
Embase	('nonalcoholic fatty liver'/exp OR 'fatty liver'/exp OR 'non-alcoholic fatty liver disease':ti,ab,kw OR 'nonalcoholic fatty liver disease':ti,ab,kw OR nafld:ti,ab,kw OR 'metabolic dysfunction-associated steatotic liver disease':ti,ab,kw OR masld:ti,ab,kw OR 'metabolic dysfunction-associated fatty liver disease':ti,ab,kw OR mafld:ti,ab,kw OR 'hepatic steatosis':ti,ab,kw OR 'fatty liver':ti,ab,kw OR 'steatotic liver disease':ti,ab,kw) AND ('atrial fibrillation'/exp OR 'heart arrhythmia'/exp OR 'atrial fibrillation':ti,ab,kw OR af:ti,ab,kw OR arrhythmia*:ti,ab,kw)
Scopus	TITLE-ABS-KEY("non-alcoholic fatty liver disease" OR "nonalcoholic fatty liver disease" OR NAFLD OR "metabolic dysfunction-associated steatotic liver disease" OR MASLD OR "metabolic dysfunction-associated fatty liver disease" OR "metabolic associated fatty liver disease" OR MAFLD OR "hepatic steatosis" OR "fatty liver" OR "steatotic liver disease") AND TITLE-ABS-KEY("atrial fibrillation" OR AF OR arrhythmia*)
Web of Science	TS=("non-alcoholic fatty liver disease" OR "nonalcoholic fatty liver disease" OR NAFLD OR "metabolic dysfunction-associated steatotic liver disease" OR MASLD OR "metabolic dysfunction-associated fatty liver disease" OR "metabolic associated fatty liver disease" OR MAFLD OR "hepatic steatosis" OR "fatty liver" OR "steatotic liver disease") AND TS=("atrial fibrillation" OR AF OR arrhythmia*)
